# Supplementary figures and images for: 16S rRNA gene amplicon-based metagenomic analysis of bacterial communities in the rhizospheres of selected mangrove species from Mida Creek and Gazi Bay, Kenya
Source: PLoS One. 2021 Mar 23;16(3):e0248485. doi: 10.1371/journal.pone.0248485 (PMC7987175; doi:10.1371/journal.pone.0248485)

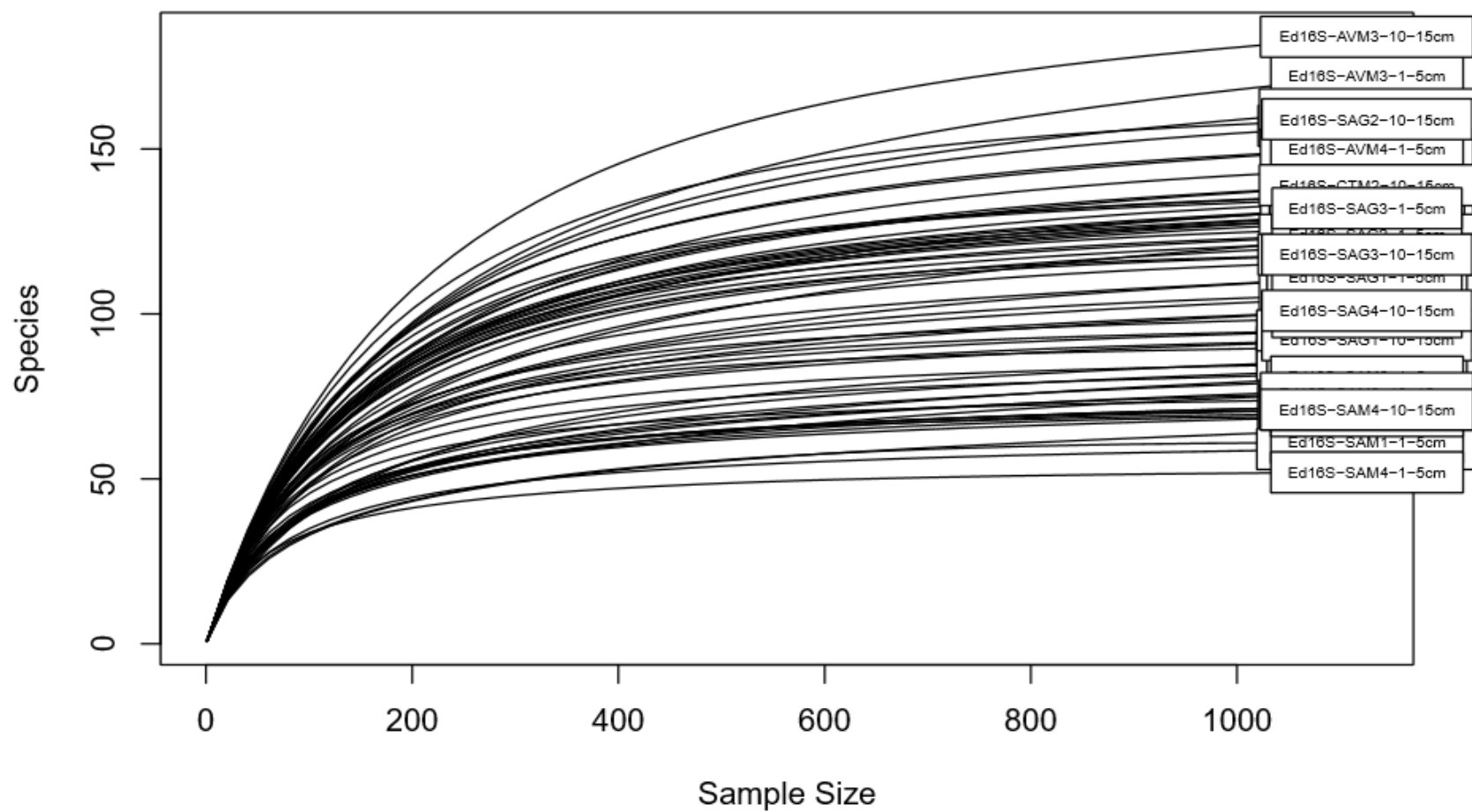

Supplement: S1 Fig — (PDF) [file pone.0248485.s001.pdf]

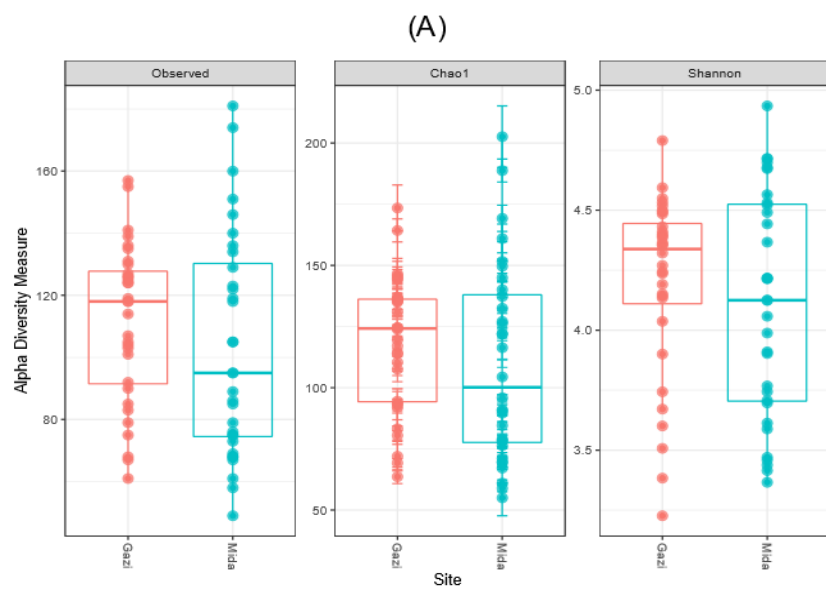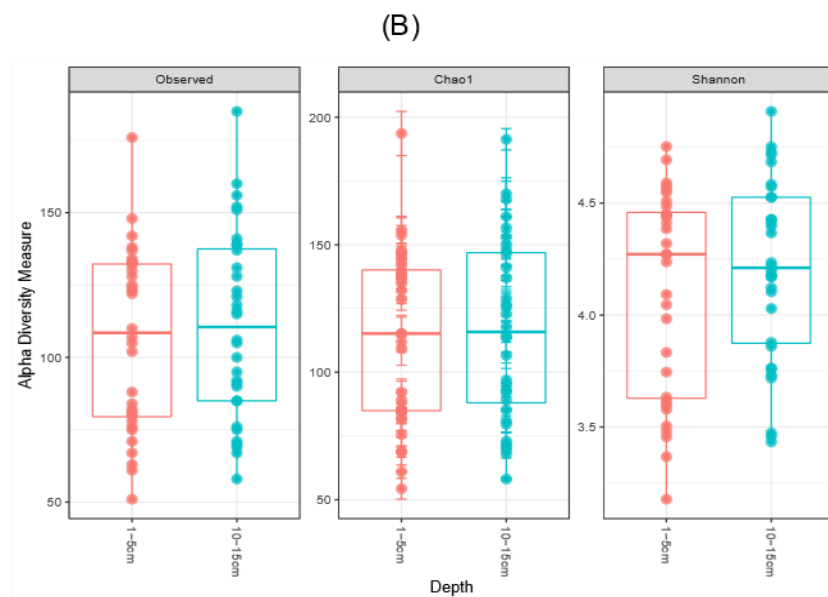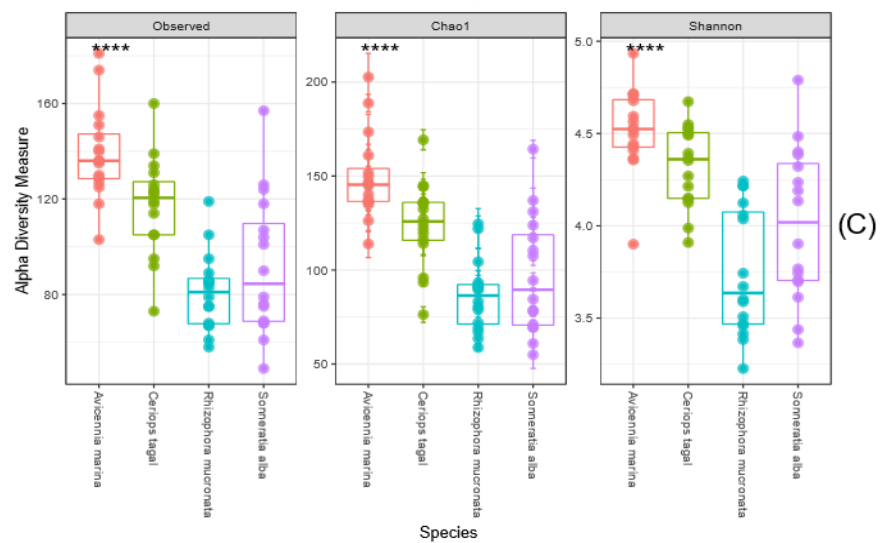

Supplement: S3 Fig — Alpha diversity differentiation based on site (A), depth (B) and mangrove species (C). (PDF) [file pone.0248485.s003.pdf]

Samples

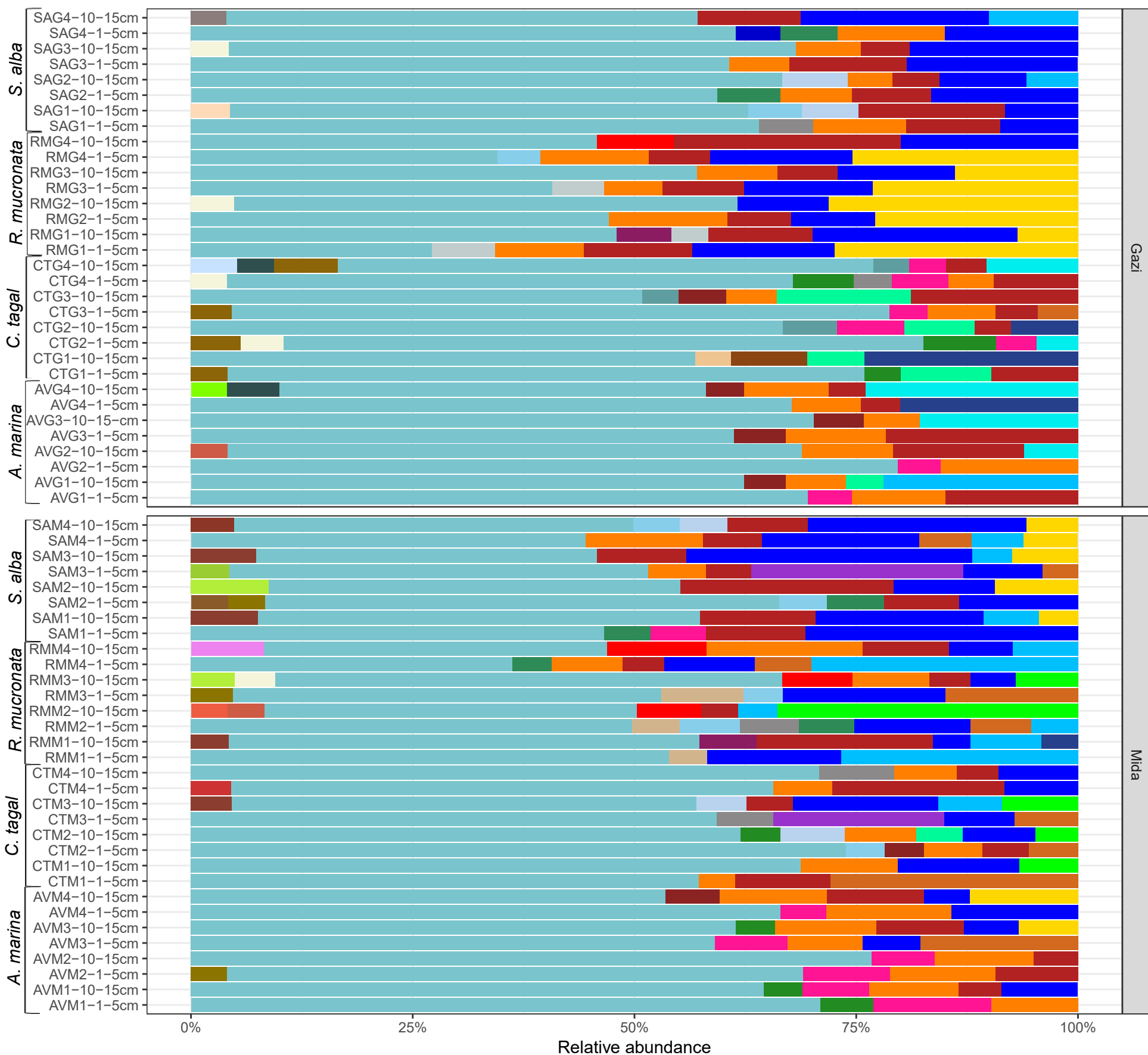

Supplement: S4 Fig — (PDF) [file pone.0248485.s004.pdf]
