# Supplementary material for: 16S rRNA gene amplicon-based metagenomic analysis of bacterial communities in the rhizospheres of selected mangrove species from Mida Creek and Gazi Bay, Kenya
Source: PLoS One. 2021 Mar 23;16(3):e0248485. doi: 10.1371/journal.pone.0248485 (PMC7987175; doi:10.1371/journal.pone.0248485)
Supplement: S2 Fig — Venn diagram of shared OTUs between the same mangrove species in different sites (A) and overall shared OTUs across both study sites (B). (PDF) [file pone.0248485.s002.pdf]

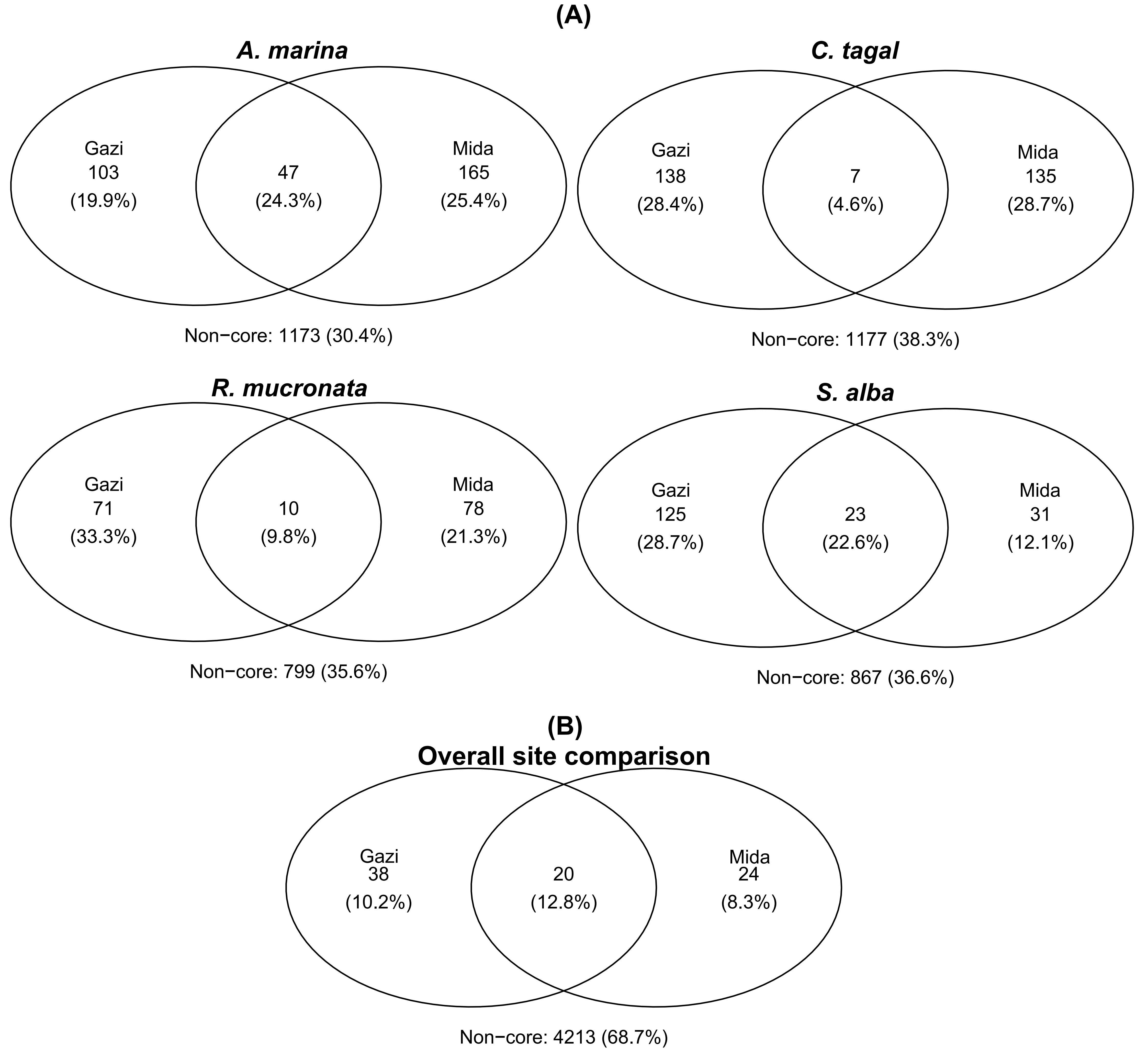
(A)
A. marina
Gazi
103
(19.9%)
47
(24.3%)
Mida
165
(25.4%)
Non−core: 1173 (30.4%)
C. tagal
Gazi
138
(28.4%)
7
(4.6%)
Mida
135
(28.7%)
Non−core: 1177 (38.3%)
R. mucronata
Gazi
71
(33.3%)
10
(9.8%)
Mida
78
(21.3%)
Non−core: 799 (35.6%)
S. alba
Gazi
125
(28.7%)
23
(22.6%)
Mida
31
(12.1%)
Non−core: 867 (36.6%)
(B)
Overall site comparison
Gazi
38
(10.2%)
20
(12.8%)
Mida
24
(8.3%)
Non−core: 4213 (68.7%)
