# Supplementary material for: 16S rRNA gene amplicon-based metagenomic analysis of bacterial communities in the rhizospheres of selected mangrove species from Mida Creek and Gazi Bay, Kenya
Source: PLoS One. 2021 Mar 23;16(3):e0248485. doi: 10.1371/journal.pone.0248485 (PMC7987175; doi:10.1371/journal.pone.0248485)
Supplement: S1 Table — (PDF) [file pone.0248485.s005.pdf]

	Ca	C	EC	Mg	N	P	K	Salinity	Na	pH
Observed	-0.3**	-0.38***	-0.23	-0.39***	-0.24*	-0.2	-0.34**	-0.23	-0.16	0.02
Chao1	-0.31**	-0.37**	-0.2	-0.38***	-0.23	-0.19	-0.33**	-0.21	-0.15	0.01
Shannon	-0.31**	-0.46***	-0.34**	-0.46***	-0.33**	-0.26*	-0.42***	-0.35**	-0.26*	0.01

(Significance codes: 0 '***' 0.001 '**' 0.01 '*' 0.05)
