# Supplementary material for: 16S rRNA gene amplicon-based metagenomic analysis of bacterial communities in the rhizospheres of selected mangrove species from Mida Creek and Gazi Bay, Kenya
Source: PLoS One. 2021 Mar 23;16(3):e0248485. doi: 10.1371/journal.pone.0248485 (PMC7987175; doi:10.1371/journal.pone.0248485)
Supplement: S2 Table — (PDF) [file pone.0248485.s006.pdf]

ID	Median clr (10-15cm)	Median clr (1-5cm)	Diff. Between	p-value	FDR-adjusted p-value
A. marina **(Gazi bay)**					
Desulfobacter	4.31	-0.85	3.00	0.04	0.82
A. marina **(Mida creek)**					
Bacteriap25 (*Myxococcales*)	5.36	-1.84	2.67	0.01	0.81
C. tagal **(Gazi bay)**					
Uncultured *Pirellulaceae*	-1.27	6.60	7.76	0.01	0.40
Uncultured *Rhodobacteraceae*	-1.25	6.41	7.60	0.01	0.39
MND1 (*Nitrosomonadaceae*)	-1.36	6.19	7.47	0.01	0.46
Candidatus Udaeobacter	-1.19	5.22	6.33	0.02	0.46
C. tagal **(Mida creek)**					
Mycobacterium	5.70	6.60	0.95	0.05	0.54
Draconibacterium	5.53	-1.38	-6.97	0.02	0.42
Uncultured *Cyclobacteriaceae*	-0.63	5.69	6.32	0.03	0.53
Sulfurimonas	6.18	-0.19	-6.53	0.03	0.52
Psychrilyobacter	7.25	-1.08	2.40	0.01	0.30
Sva0081 sediment group (*Desulfobacteraceae*)	6.49	2.77	-3.93	0.04	0.48
R. mucronate **(Gazi bay)**					
Sulfurimonas	6.06	-1.23	-7.36	0.01	0.47
Uncultured *Rhodospirillales*	-0.43	6.49	6.95	0.04	0.80
R. mucronate **(Mida creek)**					
Robiginitalea	-0.03	5.98	3.72	0.05	0.53
Fusibacter	6.59	-0.26	-6.81	0.04	0.51
Psychrilyobacter	6.60	-1.12	-8.10	0.01	0.31
Desulfatiglans	8.35	1.30	-6.76	0.04	0.52
Woeseia	-1.01	7.05	7.79	0.01	0.26
S. alba **(Gazi bay)**					
Uncultured *Microcystaceae*	-1.75	5.19	7.75	0.01	0.65
S. alba **(Mida creek)**					

Subgroup 10 (*Thermoanaerobaculaceae*)	-0.07	6.03	6.29	0.03	0.54
Uncultured (*Thiotrichaceae*)	-0.05	6.36	6.52	0.02	0.60
Chthoniobacter	0.16	5.48	5.39	0.05	0.66
