# Supplementary material for: 16S rRNA gene amplicon-based metagenomic analysis of bacterial communities in the rhizospheres of selected mangrove species from Mida Creek and Gazi Bay, Kenya
Source: PLoS One. 2021 Mar 23;16(3):e0248485. doi: 10.1371/journal.pone.0248485 (PMC7987175; doi:10.1371/journal.pone.0248485)
Supplement: S3 Table — (PDF) [file pone.0248485.s007.pdf]

	Df	Sum of squares	R^2	F	Pr(>F)
**Species**					
	3	4.3531	0.15799	3.7526	0.001***
Residual	60	23.2	0.84201		
Total	63	27.553	1		
**Depth**					
	2	1.1213	0.04069	1.2938	0.017*
Residual	61	26.4318	0.95931		
Total	63	27.553	1		
**Site**					
	1	1.227	0.04453	2.8898	0.001***
Residual	62	26.326	0.95547		
Total	63	27.553	1		
