# Supplementary material for: 16S rRNA gene amplicon-based metagenomic analysis of bacterial communities in the rhizospheres of selected mangrove species from Mida Creek and Gazi Bay, Kenya
Source: PLoS One. 2021 Mar 23;16(3):e0248485. doi: 10.1371/journal.pone.0248485 (PMC7987175; doi:10.1371/journal.pone.0248485)
Supplement: S4 Table — (PDF) [file pone.0248485.s008.pdf]

S/N	*Comparison*	R^2	F	df	p	p.adj
1	*Avicennia marina* vs *Ceriops tagal*	0.098609	3.281892	1;30	0.001	0.001
2	*Avicennia marina* vs *Rhizophora mucronata*	0.147014	5.170564	1;30	0.001	0.001
3	*Avicennia marina* vs *Sonneratia alba*	0.146787	5.161199	1;30	0.001	0.001
4	*Ceriops tagal* vs *Rhizophora mucronata*	0.095583	3.170542	1;30	0.001	0.001
5	*Ceriops tagal* vs *Sonneratia alba*	0.103096	3.448407	1;30	0.001	0.001
6	*Rhizophora mucronata* vs *Sonneratia alba*	0.0757	2.457003	1;30	0.001	0.001
