# Supplementary material for: 16S rRNA gene amplicon-based metagenomic analysis of bacterial communities in the rhizospheres of selected mangrove species from Mida Creek and Gazi Bay, Kenya
Source: PLoS One. 2021 Mar 23;16(3):e0248485. doi: 10.1371/journal.pone.0248485 (PMC7987175; doi:10.1371/journal.pone.0248485)
Supplement: S5 Table — (PDF) [file pone.0248485.s009.pdf]

	**Correlation coefficient (*p*)**									
**Genus**	**Calcium**	**Carbon**	**EC**	**Magnesium**	**Nitrogen**	**Phosphorus**	**Potassium**	**Salinity**	**Sodium**	**pH**
Sva0081 sediment group	0.12	0.03	0.05	0.01	-0.12	-0.01	-0.18	0.05	-0.2	-0.18
Sulfurimonas	-0.14	-0.19	-0.03	-0.2	-0.19	-0.21	-0.14	-0.03	-0.16	-0.21
Arcobacter	-0.1	-0.09	-0.07	-0.12	-0.03	0.04	-0.11	-0.07	-0.07	0.19
Pir4 lineage	-0.28*	0.3**	0.46***	0.16	0.37***	0.16	0.48***	0.46***	0.45***	-0.28**
Woeseia	0.26*	0.18	-0.06	0.24	0.14	0.22	-0.03	-0.06	0.09	0.18
Blastopirellula	-0.02	0.21	0.21	0.11	0.07	0.08	0.23	0.21	0.08	-0.25*
wb1 A12	-0.19	-0.21	-0.02	-0.2	-0.17	-0.16	-0.04	-0.03	-0.03	0.13
Spirochaeta	0.15	-0.16	-0.16	-0.1	-0.25*	-0.23	-0.21	-0.16	-0.22	0.02
Vibrio	0.35***	0.08	-0.03	0.16	-0.01	0.03	-0.1	-0.03	0.01	0.31**
Psychrilyobacter	-0.06	-0.12	-0.17	-0.12	-0.11	-0.17	-0.01	-0.17	-0.1	-0.07
Mycobacterium	0.23	0.1	-0.08	0.17	-0.03	0.07	-0.13	-0.08	-0.04	-0.04
Desulfobacter	0.41***	0.23	0.03	0.3**	0.1	0.19	-0.05	0.03	0.05	0.24*
Sulfurovum	0.16	0	-0.09	0.08	-0.04	0.07	-0.07	-0.09	-0.03	0.15
Pleurocapsa PCC.7319	0.32**	0.23	0.05	0.33**	0.14	0.25*	0.13	0.05	0.15	0.01
Rhodopirellula	-0.19	0.21	0.26*	0.09	0.27*	0.21	0.25*	0.26*	0.26*	-0.12
Desulfatiglans	-0.25*	-0.17	-0.09	-0.24	-0.17	-0.23	-0.09	-0.09	-0.17	-0.37**
Desulfatitalea	0.19	0.08	-0.02	0.13	0	-0.02	0.01	-0.02	0	0.16
SEEP.SRB1	0.15	-0.02	0	0.03	-0.1	-0.06	-0.09	-0.01	-0.11	0.06
Draconibacterium	-0.05	0.03	0.2	-0.01	0.03	-0.11	0.03	0.2	0.14	-0.05
Marinobacterium	-0.2	0.08	0.27*	-0.03	0.09	0.02	0.22	0.26*	0.15	-0.3**
