# Supplementary material for: 16S rRNA gene amplicon-based metagenomic analysis of bacterial communities in the rhizospheres of selected mangrove species from Mida Creek and Gazi Bay, Kenya
Source: PLoS One. 2021 Mar 23;16(3):e0248485. doi: 10.1371/journal.pone.0248485 (PMC7987175; doi:10.1371/journal.pone.0248485)
Supplement: S6 Table — (PDF) [file pone.0248485.s010.pdf]

Table S6: Complete list of biomarkers for energy metabolism and the biosynthesis of secondary metabolites detected among mangrove species in Gazi Bay

S/N	KEGG-LEVEL III	KOs	Specie	LDA Score	p-value (FDR-adjusted)
**A.**	**Energy Metabolism**		*Sonneratia alba*	3.313678	2.25E-05
1.	Carbon fixation pathways in prokaryotes		*Rhizophora mucronata*	3.405439	1.04E-05
	Carbon fixation pathways in prokaryotes	K00174	Rhizophora mucronata	2.623394	2.62E-05
	Carbon fixation pathways in prokaryotes	K00175	*Rhizophora mucronata*	2.558543	2.66E-05
	Carbon fixation pathways in prokaryotes	K00177	*Rhizophora mucronata*	2.549422	8.21E-06
	Carbon fixation pathways in prokaryotes	K00176	*Rhizophora mucronata*	2.548897	7.71E-06
	Carbon fixation pathways in prokaryotes	K00626	*Rhizophora mucronata*	2.430842	8.07E-05
	Carbon fixation pathways in prokaryotes	K01007	*Sonneratia alba*	2.272777	2.18E-05
	Carbon fixation pathways in prokaryotes	K00198	*Rhizophora mucronata*	2.260466	3.85E-06
	Carbon fixation pathways in prokaryotes	K01963	*Avicennia marina*	2.259811	6.68E-06
	Carbon fixation pathways in prokaryotes	K15023	*Rhizophora mucronata*	2.25297	2.31E-06
	Carbon fixation pathways in prokaryotes	K05299	*Rhizophora mucronata*	2.243871	6.66E-06
	Carbon fixation pathways in prokaryotes	K01962	*Avicennia marina*	2.204808	9.92E-06
	Carbon fixation pathways in prokaryotes	K14534	*Rhizophora mucronata*	2.199343	8.74E-06
	Carbon fixation pathways in prokaryotes	K01681	*Avicennia marina*	2.171571	8.32E-06
	Carbon fixation pathways in prokaryotes	K01960	*Rhizophora mucronata*	2.170375	5.95E-05
	Carbon fixation pathways in prokaryotes	K01902	*Avicennia marina*	2.142212	1.92E-05
	Carbon fixation pathways in prokaryotes	K01903	*Avicennia marina*	2.141236	2.04E-05
	Carbon fixation pathways in prokaryotes	K00197	*Rhizophora mucronata*	2.125162	2.53E-06
	Carbon fixation pathways in prokaryotes	K00194	*Rhizophora mucronata*	2.123068	2.48E-06
	Carbon fixation pathways in prokaryotes	K01961	*Avicennia marina*	2.116454	2.16E-05
	Carbon fixation pathways in prokaryotes	K14138	*Rhizophora mucronata*	2.113725	2.48E-06
	Carbon fixation pathways in prokaryotes	K02160	*Avicennia marina*	2.042969	2.38E-05
	Carbon fixation pathways in prokaryotes	K01006	*Avicennia marina*	2.040525	2.16E-05
	Carbon fixation pathways in prokaryotes	K00031	*Ceriops tagal*	2.037342	1.68E-05
	Carbon fixation pathways in prokaryotes	K13788	*Sonneratia alba*	2.013977	4.27E-05

Table S6 Continued

S/N	KEGG-LEVEL III	KOs	Specie	LDA Score	p-value (FDR-adjusted)
2.	Methane metabolism		*Rhizophora mucronata*	3.403455	4.50E-06
	Methane metabolism	K03388	*Rhizophora mucronata*	2.822123	3.57E-06
	Methane metabolism	K00123	*Rhizophora mucronata*	2.690879	7.17E-06
	Methane metabolism	K03389	*Rhizophora mucronata*	2.503848	4.08E-06
	Methane metabolism	K11261	*Rhizophora mucronata*	2.408601	2.14E-06
	Methane metabolism	K01007	*Sonneratia alba*	2.272777	2.18E-05
	Methane metabolism	K00198	*Rhizophora mucronata*	2.260466	3.85E-06
	Methane metabolism	K05299	*Rhizophora mucronata*	2.243871	6.66E-06
	Methane metabolism	K00058	*Avicennia marina*	2.179757	0.000149
	Methane metabolism	K00197	*Rhizophora mucronata*	2.125162	2.53E-06
	Methane metabolism	K00194	*Rhizophora mucronata*	2.123068	2.48E-06
	Methane metabolism	K00441	*Sonneratia alba*	2.032775	4.44E-06
	Methane metabolism	K13788	*Sonneratia alba*	2.013977	4.27E-05
3.	Nitrogen metabolism		*Sonneratia alba*	2.616831	0.002376
	Nitrogen metabolism	K01915	*Ceriops tagal*	2.212953	1.58E-05
	Nitrogen metabolism	K05601	*Rhizophora mucronata*	2.212175	3.38E-05
	Nitrogen metabolism	K00261	*Avicennia marina*	2.031022	0.000323
	Nitrogen metabolism	K01673	*Ceriops tagal*	2.008106	0.001195
4.	Oxidative phosphorylation		*Ceriops tagal*	3.393506	2.89E-05
	Oxidative phosphorylation	K00335	*Rhizophora mucronata*	2.363296	4.37E-06
	Oxidative phosphorylation	K00334	*Rhizophora mucronata*	2.351052	7.40E-06
	Oxidative phosphorylation	K03885	*Sonneratia alba*	2.325294	9.87E-06
	Oxidative phosphorylation	K05575	*Sonneratia alba*	2.324346	0.00015
	Oxidative phosphorylation	K00341	*Ceriops tagal*	2.302181	1.10E-05
	Oxidative phosphorylation	K00342	*Avicennia marina*	2.296613	0.00018
	Oxidative phosphorylation	K00331	*Ceriops tagal*	2.286336	1.17E-05
	Oxidative phosphorylation	K00330	*Avicennia marina*	2.269924	9.68E-06

Table S6 Continued

S/N	KEGG-LEVEL III	KOs	Specie	LDA Score	p-value (FDR-adjusted)
	Oxidative phosphorylation	K00340	*Avicennia marina*	2.253945	9.79E-06
	Oxidative phosphorylation	K00332	*Avicennia marina*	2.2404	1.66E-05
	Oxidative phosphorylation	K00339	*Ceriops tagal*	2.232649	1.13E-05
	Oxidative phosphorylation	K00343	*Ceriops tagal*	2.218872	1.13E-05
	Oxidative phosphorylation	K00333	*Avicennia marina*	2.212623	1.53E-05
	Oxidative phosphorylation	K00338	*Avicennia marina*	2.207849	1.19E-05
	Oxidative phosphorylation	K00337	*Avicennia marina*	2.207471	1.12E-05
	Oxidative phosphorylation	K00336	*Ceriops tagal*	2.180692	6.54E-06
	Oxidative phosphorylation	K05577	*Sonneratia alba*	2.133053	0.000166
	Oxidative phosphorylation	K00937	*Ceriops tagal*	2.086027	2.70E-05
	Oxidative phosphorylation	K02109	*Rhizophora mucronata*	2.036367	7.00E-05
	Oxidative phosphorylation	K02259	*Ceriops tagal*	2.030212	0.000298
	Oxidative phosphorylation	K02274	*Ceriops tagal*	2.023575	0.000146
5.	Photosynthesis		*Sonneratia alba*	3.415392	0.000152
	Photosynthesis	K02639	*Sonneratia alba*	2.442877	0.000157
	Photosynthesis	K02703	*Sonneratia alba*	2.151732	0.000236
	Photosynthesis	K02720	*Sonneratia alba*	2.135218	0.000114
	Photosynthesis	K02109	*Rhizophora mucronata*	2.036367	7.00E-05
6.	Photosynthesis antenna proteins		*Sonneratia alba*	2.916338	0.000138
	Photosynthesis antenna proteins	K02290	*Sonneratia alba*	2.136482	0.000109
7.	Sulfur metabolism		*Ceriops tagal*	2.623216	0.016708
	Sulfur metabolism	K00184	*Rhizophora mucronata*	2.321493	5.79E-05
	Sulfur metabolism	K00185	*Rhizophora mucronata*	2.308368	3.56E-05
	Sulfur metabolism	K00958	*Rhizophora mucronata*	2.270879	7.61E-06
	Sulfur metabolism	K00395	*Rhizophora mucronata*	2.136051	2.73E-06
	Sulfur metabolism	K00394	*Rhizophora mucronata*	2.136051	2.73E-06
	Sulfur metabolism	K11181	*Rhizophora mucronata*	2.135135	2.95E-06

Table S6 Continued

S/N	KEGG-LEVEL III	KOs	Specie	LDA Score	p-value (FDR-adjusted)
	Sulfur metabolism	K11180	*Rhizophora mucronata*	2.135135	2.95E-06
	Sulfur metabolism	K17218	*Sonneratia alba*	2.102103	4.84E-05
	Sulfur metabolism	K01738	*Avicennia marina*	2.056735	0.000126
	Sulfur metabolism	K00390	*Ceriops tagal*	2.05207	3.94E-05
	Sulfur metabolism	K08352	*Rhizophora mucronata*	2.019324	1.30E-05
**B.**	**Biosynthesis of other secondary metabolites**		*Ceriops tagal*	3.313678	2.25E-05
1.	Acarbose and validamycin biosynthesis		*Ceriops tagal*	2.223684	3.27E-05
	Acarbose and validamycin biosynthesis	K01092	*Ceriops tagal*	2.015396	2.90E-05
2.	Monobactam biosynthesis		*Ceriops tagal*	2.670305	2.89E-05
	Monobactam biosynthesis	K00812	*Avicennia marina*	2.372198	2.16E-05
	Monobactam biosynthesis	K00208	*Rhizophora mucronata*	2.270879	7.61E-06
3.	Novobiocin biosynthesis		*Rhizophora mucronata*	2.099116	4.66E-06
	Novobiocin biosynthesis	K01714	*Ceriops tagal*	2.641063	6.71E-06
	Novobiocin biosynthesis	K00958	*Avicennia marina*	2.302526	8.17E-06
	Novobiocin biosynthesis	K00845	*Ceriops tagal*	2.141723	1.13E-05
	Novobiocin biosynthesis	K11358	*Ceriops tagal*	2.108883	1.19E-05
4.	Phenazine biosynthesis	K01710	*Avicennia marina*	2.093163	6.16E-05
	Phenylpropanoid biosynthesis		*Ceriops tagal*	2.205743	0.001174
	Phenylpropanoid biosynthesis	K04517	*Ceriops tagal*	2.182336	0.001809
	Prodigiosin biosynthesis	K00059	*Avicennia marina*	2.806895	1.59E-05
	Prodigiosin biosynthesis		*Avicennia marina*	2.676803	9.91E-06
5.	Streptomycin biosynthesis		*Ceriops tagal*	2.674609	1.05E-05
	Streptomycin biosynthesis	K00817	*Ceriops tagal*	2.121348	2.21E-05
	Streptomycin biosynthesis	K01710	*Ceriops tagal*	2.015396	2.90E-05
	Streptomycin biosynthesis	K01092	*Ceriops tagal*	2.002665	1.63E-05
