# Supplementary material for: 16S rRNA gene amplicon-based metagenomic analysis of bacterial communities in the rhizospheres of selected mangrove species from Mida Creek and Gazi Bay, Kenya
Source: PLoS One. 2021 Mar 23;16(3):e0248485. doi: 10.1371/journal.pone.0248485 (PMC7987175; doi:10.1371/journal.pone.0248485)
Supplement: S7 Table — (PDF) [file pone.0248485.s011.pdf]

Table S7: Complete list of biomarkers for energy metabolism and the biosynthesis of secondary metabolites detected among mangrove species in Mida Creek

S/N	KEGG-LEVEL3	KOs	Species	LDA Score	p-value (FDR-adjusted)
**A.**	**Carbon fixation pathways in prokaryotes**		*Sonneratia alba*	3.25015	0.000264
1.	Carbon fixation pathways in prokaryotes	K01007	*Sonneratia alba*	2.188279	0.000173
	Carbon fixation pathways in prokaryotes	K01681	*Avicennia marina*	2.048338	0.00246
	Carbon fixation pathways in prokaryotes	K01961	*Avicennia marina*	2.047994	0.001609
	Carbon fixation pathways in prokaryotes	K01902	*Avicennia marina*	2.020236	0.002038
	Carbon fixation pathways in prokaryotes	K01903	*Avicennia marina*	2.012865	0.002788
	Carbon fixation pathways in prokaryotes	K01682	*Rhizophora mucronata*	2.001052	0.000234
2.	Methane metabolism	K03388	*Sonneratia alba*	2.440457	0.004265
	Methane metabolism	K00058	*Avicennia marina*	2.222501	0.000103
	Methane metabolism	K01007	*Sonneratia alba*	2.188279	0.000173
	Methane metabolism	K00441	*Sonneratia alba*	2.003694	0.000649
3.	Nitrogen metabolism		*Sonneratia alba*	2.495576	0.002513
	Nitrogen metabolism	K00368	*Avicennia marina*	2.341172	0.006068
	Nitrogen metabolism	K01915	*Avicennia marina*	2.136372	0.001231
	Nitrogen metabolism	K05601	*Sonneratia alba*	2.055445	0.001641
4.	Oxidative phosphorylation		*Avicennia marina*	3.194153	0.001579
	Oxidative phosphorylation	K03888	*Avicennia marina*	2.340116	0.003081
	Oxidative phosphorylation	K03886	*Ceriops tagal*	2.241562	0.000696
	Oxidative phosphorylation	K05575	*Sonneratia alba*	2.218018	0.001802
	Oxidative phosphorylation	K00342	*Avicennia marina*	2.174668	0.001377
	Oxidative phosphorylation	K00330	*Avicennia marina*	2.162385	0.000868
	Oxidative phosphorylation	K00340	*Avicennia marina*	2.147791	0.001126
	Oxidative phosphorylation	K00341	*Avicennia marina*	2.137916	0.001416
	Oxidative phosphorylation	K00338	*Avicennia marina*	2.127536	0.001805
	Oxidative phosphorylation	K00332	*Avicennia marina*	2.124206	0.001334
	Oxidative phosphorylation	K00331	*Avicennia marina*	2.121745	0.001426
	Oxidative phosphorylation	K02275	*Avicennia marina*	2.075718	0.000194

Table S7 Continued

S/N	KEGG-LEVEL3	KOs	Specie	LDA Score	p-value (FDR-adjusted)
	Oxidative phosphorylation	K00339	*Avicennia marina*	2.064256	0.003168
	Oxidative phosphorylation	K00343	*Avicennia marina*	2.060812	0.002533
	Oxidative phosphorylation	K00333	*Avicennia marina*	2.058015	0.001715
	Oxidative phosphorylation	K00337	*Avicennia marina*	2.057106	0.002085
5.	Photosynthesis		*Sonneratia alba*	3.29856	0.002664
	Photosynthesis	K02639	*Sonneratia alba*	2.340152	0.001672
	Photosynthesis	K02703	*Sonneratia alba*	2.054111	0.002767
	Photosynthesis	K02720	*Sonneratia alba*	2.039176	0.001854
6.	Photosynthesis antenna proteins		*Sonneratia alba*	2.813242	0.001874
	Photosynthesis antenna proteins	K02290	*Sonneratia alba*	2.040833	0.001769
7.	Sulfur metabolism	K00958	*Sonneratia alba*	2.101466	0.000318
	Sulfur metabolism	K00955	*Avicennia marina*	2.008079	9.77E-05
**B.**	**Biosynthesis of other secondary metabolites**		*Avicennia marina*	3.25015	0.000264
1.	Acarbose and validamycin biosynthesis		*Avicennia marina*	2.044338	0.016839
2.	cephalosporin biosynthesis	K04127	*Avicennia marina*	2.158621	0.000253
3.	Monobactam biosynthesis		*Ceriops tagal*	2.643872	0.000797
	Monobactam biosynthesis	K01714	*Avicennia marina*	2.401851	4.31E-05
	Monobactam biosynthesis	K00958	*Sonneratia alba*	2.101466	0.000318
	Monobactam biosynthesis	K00955	*Avicennia marina*	2.008079	9.77E-05
4.	Novobiocin biosynthesis		*Avicennia marina*	2.514862	0.000219
	Novobiocin biosynthesis	K00812	*Avicennia marina*	2.213835	0.000173
5.	Phenylpropanoid biosynthesis	K05349	*Ceriops tagal*	2.003998	0.007876
6.	Prodigiosin biosynthesis		*Avicennia marina*	2.769566	0.00015
	Prodigiosin biosynthesis	K00059	*Avicennia marina*	2.643418	0.000219
	Prodigiosin biosynthesis	K00208	*Avicennia marina*	2.102357	0.001853
7.	Streptomycin biosynthesis		*Avicennia marina*	2.509342	0.006985
	Streptomycin biosynthesis	K00845	*Avicennia marina*	2.07378	0.000212
	Streptomycin biosynthesis	K01092	*Avicennia marina*	2.015111	0.006958
