# Supplementary material for: 16S rRNA gene amplicon-based metagenomic analysis of bacterial communities in the rhizospheres of selected mangrove species from Mida Creek and Gazi Bay, Kenya
Source: PLoS One. 2021 Mar 23;16(3):e0248485. doi: 10.1371/journal.pone.0248485 (PMC7987175; doi:10.1371/journal.pone.0248485)
Supplement: Attachment — Submitted filename: PONE-D-20-21986_Response to Reviewers.doc [file pone.0248485.s012.doc]

**Report on the responses raised by reviewers**

**PONE-D-20-21986**
16S rRNA gene amplicon-based metagenomic analysis of bacterial communities in the rhizospheres of selected mangrove species from Mida Creek and Gazi Bay, Kenya

***NB:*** *Sections, page numbers and line numbers mentioned below refers to the sections, page numbers and line numbers of the ‘revised manuscript with track changes.’*

**Responses to comments raised by Reviewer #1:**

1. Overall the study is well presented and a lot of work has been done. However, there is a major flaw in the experimental design, authors had only collected (multiple samples) only from one tree for each species so that the results cannot be generalized in any way.

- **Based on background information regarding mangrove species – bacterial association, significant shifts are not expected for the same mangrove species within the same location. While we agree with the reviewer that our study would have been more robust with more sampling effort covering more trees for each mangrove specie, we understand that significant insight can still be drawn from our study with the current sampling campaign.**

2. Revised the text a lot of hyperbole example myriad of bacterial communities and repetition e.g. mangrove ecosystem (3 times in 5 lines 64-68)

- **We have revised the text as highlighted in the corrected manuscript (Lines 72 – 75).**

3. Introduction: lines 70 -79 Authors refer to another studies but they simply report the main conclusion without any explanation on what they expect to find based on this results or how this study will help in their own one.

- **We have revised the section to connect its importance to our study (Lines 72 – 90).**

**Methods:**

4. Number of samples: even we the authors try to maximize the replication within the sampling campaign having only one tree for specie lead to a high uncertainty of the conclusion. Peculiar condition, maybe not notable, could been presented and the results cannot be generalized, it required to have more than one tree per specie sampled to corroborate the results.

- **Sampling more trees for each mangrove specie would have certainly made the study more robust, however, the sampling effort undertaken is sufficient to provide substantial insights considering that a drastic shift is not expected within identical mangrove species in the same location.**

5. How common are each species in the two sites? one is dominant and the remaining 3 rare or they shared the same % across the mangrove? please provide this data.

- **We have indicated on the methodology that the predominant mangrove species contribute to over 80% of the mangrove formation in both sites (Lines 140 – 142).**

6. Fig. 2 colour should not be shared between phylum and genus graph

- **Changes have been made to Fig. 2 (now Fig 1) as recommended**

7. Results: Authors refer to data as no difference in depth is present for almost all the entire Results section with the exeption of the RDA results in which the differential depth is considered. If the authors want to include this factor also the previous section should include (as supplementary or how they prefer) this differentiation and data should be analyzed accordingly.

- **Result of depth analysis have been included in supplementary materials (S2 Table)**

8. Fig. 5 need to be improved hardly readable

- **Fig. 5 (now Fig 4) has been improved as recommended**

9. Discussion:
   Authors refer across the text to their results without including reference to figure or table please amend. A big part of the discussion is devoted to a sort of list of which organism had been found but without adding any other information or discussion point, this part of the text should be cut or more discussion point should be inserted or merged with the following section about the putative pathway in microbial communities.

- **Reference to figures and Table has been included in the discussion section of the manuscript (Lines 533, 536, 547, 549, 558, 577, 606, 621)**
- **A large chunk of the discussion section describing organisms that have been found in mangrove environment has been deleted as recommended (Line 595-596, 606-612)**

**Responses to comments raised by Reviewer #2:**

In this manuscript, Muwawa and collaborators investigated the bacterial community associated to the rhizosphere of four different mangrove species, in two distinct locations, along the coast of Kenya. The two locations present a different level of anthropogenic pollution and among these sites the authors investigated different factors that can affect the bacterial community composition associated to the mangrove sediments, highlighting the mangrove species and the chemical properties of the sediments as important factors that determine the bacterial community composition in the mangrove ecosystem. I found this work interesting and well written. The authors through a combination of high-throughput sequencing data and sediment chemical analysis provided results that suggest how the root system of each mangrove species is important in determining the diversity and the composition of bacterial communities associated to mangrove sediments. However, I found some aspects that may require more information to strengthen the authors findings. I will report my comments in the next paragraphs.

- **We appreciate the positive comments from the reviewer.**

**Major issues**

1. While I am convinced about the species-specific effect of route exudate on the bacterial community composition associated to the mangrove rhizosphere, I have found along the paper some contradictory parts, especially in the discussion paragraph, between the importance of location and nutrients concentration. The authors showed that the geographical location significantly affected the diversity of the bacterial community, also if it is not the main affecting factor. Authors also stated the importance of the chemical composition of the sediments, since Mida creek is characterized by a higher concentration of nutrients that decrease the bacterial diversity, due to more intense anthropogenic activities. If it is possible, to clarify and better investigated which factors are more affecting the bacterial community composition, I think that the authors should consider to include in the analysis bulk soil samples as control, collected far from the plant roots in the two sites, to have a comparison of the bacterial community and the chemical composition associated to the sediment not affected by the mangrove root system. In this way it should be possible to consider if there are variations in the bacterial community composition that are due to differences in the sediment characteristics, that are not influenced by the presence of the different mangroves species and which parameters could be affected by anthropogenic activities.

- **We agree with the reviewer comments on clarifying the parameters that influence the bacterial community composition within the mangroves. However, the suggestion stated (analysis of bulk soil samples as control) was not considered during the study design, hence the bulk soil samples were not analyzed. This can be considered in future comprehensive mangrove microbial studies.**
- **Contradiction on the importance of geolocation and physicochemical parameters have been addressed (Lines 533 – 534 and 547 – 556). Specifically, statements indicating that geolocation did not influence bacterial alpha diversity have been modified. These two parameters are intertwined since the main differences between the two study sites is the higher nutrient influx in Mida creek due to anthropogenic activities. Besides, there were significant differences in alpha diversity between same mangrove species found in the different sites (Table 1)**

2. (a). In general, I think that the authors should give more information regarding some aspects of the experiments, some also linked to my previous comment. The authors initially explained that the two sites along the Kenyan coast have been chosen due to the different level of pollution in the two areas due to anthropogenic activities, but they never mentioned which are the main parameters that could be affected in the mangrove ecosystems. Moreover, when the authors compared the two sites, they described them using different parameters. For instance, for Mida creek they provided information on the temperature range and the range of rain precipitation, while for Gazi Bay they provided the average temperature and humidity. If it is possible, I suggest that the parameters adopted to described the areas would be the same (line 110-117).

- **We have made some changes in the text and described the parameters (temperature and humidity) in both sites so that there is uniformity (Lines 124 – 139). The anthropogenic activities that are prevalent at Mida creek site have been described and are thought to cause pollution in the area that may influence the bacterial community composition (Lines 103 – 106, 130-133).**

(b) In line 117-121 Authors gave a general overview on how the four mangrove species are distributed in Kenyan mangrove forest, but they don’t provide details on the distribution that each species covers in the two studied areas. Is the distribution and the proportion of the four species different in the two locations? Where the different three were collected? in the seaward side or in the middle zone? Was the tide affecting differently the mangroves sampled in the two location or at the different depths considered? Maybe the authors could provide more information on the experimental design to better describe the mangroves in the two sampling sites and exclude that other environmental factors could have affected the bacterial community composition in the rhizosphere beside the species selection effect of the plants.

- **We have made some changes in the methodology to describe the mangrove zonation (distribution of the mangrove species, especially the predominant species) which takes the same pattern in both sites as indicated in the text. The details of mangrove species distribution whether seaward, in the middle or landward is indicated (Lines 140 - 153).**
- **We have also pointed out in the text the temperature and tides conditions during sampling as highlighted in the manuscript text (Lines 160-161).**

(c). Were the temperature and tide level homogeneous in the sampling sites for all the samples or there were significant variations in these parameters? These variables are usually very important and can deeply affect the bacterial community composition in mangrove sediments. May the authors comment if these parameters have been taken in consideration?

- **We have indicated in the methodology the conditions during our sampling in the two sites. There was no significant variation in the parameters (temperature and tides) stated (Lines 160 – 161). Sampling was done during low tides at both sites. Mida creek had a temperature of 27°C, while Gazi bay had 28°C.**

3. The functional predictions of the mangrove sediment bacterial community from 16S rRNA amplicon data has to be consider as a first overview of the bacterial community metabolic potential. The data obtained are good for the purpose of this manuscript but further studies need to investigate more in details these results, in future works. Due to the low resolution at low taxonomic level of amplicon data, the prediction could be affected by substantial bias that metagenomic or metatranscriptomic analysis can resolve.

- **We agree with the reviewer that the functional prediction is a first overview and have thus recommended the application multi-omics techniques in future investigations (Lines 669 – 676)**

**Minor issues**

1. In Figure S2 to highlight the importance of the plants on the bacterial community composition in the rhizosphere, could be more useful to see how many OTUs are in common between the same mangrove species in the 2 different locations. May the author add a Venn diagram panel also with this comparison?
   I also found strange that Gazy bay does not have any OTU not shared with Mida creek. The addition of this panel may immediately give an idea on how many OTUs are shared between the rhizosphere of the same mangrove species in the two different locations, strengthen the importance of the species-specific plant effect in the selection of bacterial communities associated to the rhizosphere independently by the geographical location.

- **A venn diagram panel comparing core OTUs in each mangrove species in both sites have been included as recommended (S2 Fig). The relevant results section has also been updated (Lines 264-271)**

2. Probably in line 241 the authors are referring to Figure S3 panel A instead C.

- **Yes, we were referring to figure S3A. The required correction has been affected in the revised manuscript (Lines 280-285)**

3. In Figure 2 panel B I would suggest to align in the graph the relative abundance of the genus less abundant than 5%. In this way would be easier for the reader to appreciate the differences between the genus that the authors took in consideration in the analysis.

- **The percentage of less abundant genera have been included to the legend of Figure 2B (now Fig 1B) (Line 328 – 330).**

4. I suggest to add the taxonomic bacterial composition at family level in the supplementary material since authors described these results between line 279 and 293.

- **The bacterial composition at family level has been added to supplementary materials (S4 Fig)**

5. In Figure 6 change the color code for the sites, black and purple are difficult to be distinguished.

- **The colour code for sites has been changed as recommended (now Fig 5)**

6. I found the paragraph from line 510 in the discussion redundant with the paragraph in the results section starting at line 265. I suggest that the authors rephrase the discussion paragraph avoiding the detail of the percentage already reported in the result section and focus more they comment more on their findings.

- **We have rephrased the discussion paragraph and removed the percentages as suggested by the reviewer. See the highlighted text in the corrected manuscript.**

**Responses to comments raised by Reviewer #3:**

1. The work is a very interesting contribute. I recommend authors to play more attention to the clarity of sentences and writing errors. Data supports authors’ discussion and conclusions. Discussion could be more rich especially in exploring diversities in microbial communities composition in the two sites (playing attention to role of pollution that is the main difference between the two sites described by authors since the very beginning) or among different species.

- **We have made changes on the clarity of sentences and some grammatical errors as highlighted in the text.**
- **Differentially abundant bacterial taxa of same mangrove species in both sites have been included in the results section (Lines 318 – 325, 352 – 369) and the corresponding discussion section has been updated (Lines 635-639)**

**Minor review**

2. Note 1: check if measure unit and negative signs are written beside numerical data with appropriate space (especially lines 113-134).

- **We have confirmed that measure units and negative signs are written beside numerical data with appropriate space.**

3. Lines 70-78: check for some errors in the language.

- **We have corrected lines 70-81 as highlighted in the text.**

4. Lines 70: I’d suggest to summarize a little bit more in details the ref (10) experimental design.

- **We have summarized the statement and made it clear.**

5. The sense of sentence at line 74-75 is not clear.

- **We have corrected the entire paragraph.**

6. Lines 82-83: the phrase “due to inadequate efforts spent in exploring the mangrove habitats for microbial diversity” is maybe too strong (especially without reference).

- **We have corrected the phrase as highlighted and given some references i.e. …... ‘However, data on microbial community diversity is limited due to low attention in exploring the mangrove habitats for microbial diversity (14,15)’**

7. Lines 94: better describe what kind of pollution resources are present in Mida Creek site.

- **We have stated the anthropogenic activities that are prevalent at the Mida creek (Lines 103 – 133 and 130-133).**

8. Lines 109-116: data of temperature and humidity are not described homogeneously for the two sites.

- **We have corrected the referred lines in the study site section and provided homogeneous data of temperature and humidity as highlighted in the text (Lines 125-139).**

9. Lines 138-141: method should be described more in details or a reference should be present.

- **We have provided references for the methods used as highlighted in the text (Lines 173 – 176).**

10. Lines 452-54 are the exact copy of lines 57-58.

- **We have corrected the referred statements in the lines (520 - 522) under the discussion section as highlighted in the text.**

11. Lines 500-524: check for typos and errors in language.

- **We have corrected the typos and errors in the referred text section as indicated in the text.**

**Major review**

12. For the description of a proper vertical distribution I’d suggest to the authors to sample at 3 different depths instead of two (done in Wu et al. 2016 – ref 10).

- **We agree with the reviewer’s comment; however, we did not factor that in during our study design, partly due to some limited funding and we can recommend for such design in a future comprehensive study on mangrove microbial communities.**

**Response to Editor’s comments:**

**Journal Requirements:**

When submitting your revision, we need you to address these additional requirements.

1. Please ensure that your manuscript meets PLOS ONE's style requirements, including those for file naming. The PLOS ONE style templates can be found at

<https://journals.plos.org/plosone/s/file?id=wjVg/PLOSOne_formatting_sample_main_body.pdf> and

<https://journals.plos.org/plosone/s/file?id=ba62/PLOSOne_formatting_sample_title_authors_affiliations.pdf>

- **We have ensured that all the PLOS One’s style requirements have been adhered to thorough the manuscript.**

2. We note that you are reporting an analysis of a microarray, next-generation sequencing, or deep sequencing data set. PLOS requires that authors comply with field-specific standards for preparation, recording, and deposition of data in repositories appropriate to their field. Please upload these data to a stable, public repository (such as ArrayExpress, Gene Expression Omnibus (GEO), DNA Data Bank of Japan (DDBJ), NCBI GenBank, NCBI Sequence Read Archive, or EMBL Nucleotide Sequence Database (ENA)). In your revised cover letter, please provide the relevant accession numbers that may be used to access these data. For a full list of recommended repositories, see http://journals.plos.org/plosone/s/data-availability#loc-omics or [http://journals.plos.org/plosone/s/data-availability#loc-sequencing](http://journals.plos.org/plosone/s/data-availability#loc-sequencing).

- **Our sequencing data have been uploaded onto the NCBI Sequence Read Archi (Ref: BioProject ID PRJNA644929).**

3. We note that [Figure 1] in your submission contain [map/satellite] images which may be copyrighted. All PLOS content is published under the Creative Commons Attribution License (CC BY 4.0), which means that the manuscript, images, and Supporting Information files will be freely available online, and any third party is permitted to access, download, copy, distribute, and use these materials in any way, even commercially, with proper attribution. For these reasons, we cannot publish previously copyrighted maps or satellite images created using proprietary data, such as Google software (Google Maps, Street View, and Earth). For more information, see our copyright guidelines: <http://journals.plos.org/plosone/s/licenses-and-copyright>.

 We require you to either (1) present written permission from the copyright holder to publish these figures specifically under the CC BY 4.0 license, or (2) remove the figures from your submission:

- **We have removed the figure to avoid the copyright issues.**
