# Supplementary material for: 16S rRNA gene amplicon-based metagenomic analysis of bacterial communities in the rhizospheres of selected mangrove species from Mida Creek and Gazi Bay, Kenya
Source: PLoS One. 2021 Mar 23;16(3):e0248485. doi: 10.1371/journal.pone.0248485 (PMC7987175; doi:10.1371/journal.pone.0248485)
Supplement: Attachment — Submitted filename: Review II.docx [file pone.0248485.s013.docx]

In my opinion the corrections applied are adequate.

The paper is now more clear and precise in form and contents.

Line 67-69: check the sentence grammar.
