# Supplementary material for: 16S rRNA gene amplicon-based metagenomic analysis of bacterial communities in the rhizospheres of selected mangrove species from Mida Creek and Gazi Bay, Kenya
Source: PLoS One. 2021 Mar 23;16(3):e0248485. doi: 10.1371/journal.pone.0248485 (PMC7987175; doi:10.1371/journal.pone.0248485)
Supplement: Attachment — Submitted filename: Responses to Reviewers.doc [file pone.0248485.s014.doc]

**Report on the responses raised by reviewers**

**PONE-D-20-21986**
16S rRNA gene amplicon-based metagenomic analysis of bacterial communities in the rhizospheres of selected mangrove species from Mida Creek and Gazi Bay, Kenya

**Responses to comments raised by Reviewer #2:**

 I found the revised version of the manuscript of Muwawa and collaborators clearly improved. The authors put a good effort to comply with the reviewers’ comments and I do not have further clarification to ask to the authors. However, I reported a very few minor comments, that I think need to be addressed before the publication.

1. In the abstract line 27, Mida Creek and Gazi Bay are both in capital letters, while in the manuscript after this point, the capital letter was removed in the second part of the name. In line 112 happened the same. Give a check and be consistent.

- **Mida Creek and Gazi Bay is now used consistently throughout the manuscript.**

2. In lines 50-52, in the brackets there is a space between the numbers and the percentage symbol. I suggest to remove it and in general to keep the same style along the all text (i.e. in line 52 there is no space).

- **Done as recommended (lines 49 – 51)**

3. In lines 125-126 “Gazy bay and Mida creek had low tides and temperature of 28°C and 27°C.” Are the authors referring at the conditions found at the time of the sampling?

Remove this sentence here, since it is out of context and it is repeated already in the collection of samples paragraph, where gives important information on the conditions in the sampling sites.

- **Yes, the stated conditions are those that were found at the time of the sampling.**
- **The sentence has been removed now.**

4. In Figure 1 panel B, (old Figure 2) my comment was misunderstood. If it is possible, I suggested to align in the graph, in panel B, the relative abundance of the genus less abundant than 4%. In this way it would be easier for the reader to appreciate the differences between the genus that the authors took in consideration in the analysis and there will not be some taxa before and after the “others” bar, like the author did in panel A.

- **Fig 1 panel B has been replaced and recommendation by reviewer has been effected.**

5. Maybe it is my mistake but I didn’t fine the Supplementary Figure S4 mentioned in line 313

- **Fig S4 is in line 319.**

6. In line 351 probably the authors are referring to figure 2 and not 3

- **Authors were referring to Figure 2.**

7. For Figure 4, there is only the caption title. A small description of the figure could help the reader to understand it.

- **A small description of the figure has been included (Lines 454 – 458)**

8. Lines 545-548, the two sentences are quite repetitive. Try to rephrase them better

- **Done as recommended (545 – 546)**

**Responses to comments raised by Reviewer #3:**

In my opinion the corrections applied are adequate.
The paper is now more clear and precise in form and contents.

1. Line 67-69: check the sentence grammar.

- **The sentence in line 67-69 has been rephrased to correct the grammar.**

**Responses to comments raised by Reviewer #4:**

1. In the manuscript titled “16S rRNA gene amplicon-based metagenomic analysis of bacterial communities in the rhizospheres of selected mangrove species from Mida Creek and Gazi Bay, Kenya” by Muwawa et al., the rhizosphere of four different mangrove species at two sites along the coast of Kenya is analysed for sedimentary conditions and the microbial community composition. The study shows how different areas within the forest dominated by different tree species contain varying bacterial communities. Furthermore, the authors identify potential drivers of bacterial community composition. This work provides novel insights into the bacterial communities of mangrove soils in eastern Africa, and adds to a growing datatbase of mangrove microbiomes across the globe. I commend the authors for their work and the neat presentation of the manuscript.

- **We appreciate the positive comments.**

I recommend however, to complete a thorough grammar-, spell-, and punctuation-check on the entire document as there were too many mistakes for me to point out.

- **We have revised and corrected the entire document for grammar, spelling and punctuation mistakes as recommended.**

I do not agree with the definition of rhizospheric sediment as it is presented in the manuscript, as it seems that the samples were taken from below the trees and with roots present in the sample, but there is no evidence that this area is actively mediated by root exudates (I would have sampled the soil directly off the roots). As there may be wider definitions of what the rhizosphere is (I am aware the methodology followed Wu et al. 2016), I would like to see it defined for this study, and the sampling method justified.

- **We have edited the sampling methods to explain the procedure of obtaining the rhizosphere sediment. The coring was performed closely to the mangrove roots in each case in order to capture the rhizosphere sediment. During the DNA extraction, the excised roots containing rhizosphere sediment were placed in a 50 mL sterile falcon tube containing autoclaved phosphate buffer and shaken for 2 min to release the rhizosphere sediment from the surface of the roots.**

Throughout the document, there is an inconsistency using R. mucronate instead of R. mucronata.

- **The name *R. mucronate* has been changed to *R. mucronate* throughout the document.**

**Abstract**

2. There is no need for specification of methods (such as which alpha diversity metrics used) and p-values in the abstract

- **Done as recommended (Lines 28 – 29, 31 – 34, 40 and 42)**

**Introduction**

3. Lines 125-126: This sentence gets repeated in the “collection of samples” section. Can be removed here.

- **The sentence in lines 125-126 has been removed.**

4. Lines 126-133: The contribution of each species is unclear: Do all four species together contribute to 80% of the mangrove forest? If yes, what is the contribution of each species? This doesn’t need to be exact, but if the microbiome varies between these stands, it would be good to know their approximate extent.

- **Yes, all the four species together contribute to 80% of the mangrove forest. The contribution of each species was not exactly determined during sampling, but it can be estimated in terms of dominance as** ***A. marina* (30%), *R. Mucronate* (25%), *S. alba* (15%) and *C. tagal* (10%).**

5. Line 142: Four trees of each species means 4x4 =16 individual trees were sampled. If this is the case, I apologise, please ignore the comment. However, I believe “one tree each of the four species was sampled”, is what was actually done.

- **Line 142 was corrected to mean ‘Four mangrove tree species’ i.e. one tree for each of the four species selected.**

6. Lines 143-145: I find the description of the sampling procedure confusing. It may help to break down the sentences as much as possible to keep each action simple and clear. A diagram of the sampling design would help immensely.

- **The sampling procedure has been revised as recommended, but we were not able to provide diagram.**

7. Line 146: When calculating the number of samples a 4x4x2x2 structure is used, while it is described in the text as 4x8x2. Keep it consistent for clarity.

- **The description in line 142 has been corrected to mean 4 mangrove tree species so as to be consistent with the 4x4x2x2 structure indicated.**

8. Line 198: I wonder, why OTUs were used for community metrics but ASVs for the functional alignment? ASVs and OTUs are two methods, that rely on very different ideas. In this way, I do not think the community data and the functional data can be compared.

- **The functional prediction has been reversed from ASVs to OTUs for uniformity (Line 200, 436 – 443, 445 - 473). Picrust2 functional prediction is based on representative sequences of either ASVs or OTUs. In our case, OTUs were clustered from ASVs and it was on this basis that representative ASV sequences was used for the prediction.**

**Results**

9. Line 351: Figure mention should be Fig. 2 instead of Fig. 3

- **Done (Line 357)**

10. Lines 394-398: The wording in these sentences doesn’t quite fit the analysis. The environmental factors are able to separate the sites/samples, ie pH separated the samples along the y axis (Gazi vs Mida). pH has an effect on the bacterial community in all sites but is here able to explain some of the differences between them.

- **The result presentation in this section has been modified to clearly reflect the influence of pH on the bacterial communities within and across study sites (Lines 400 – 402)**

11. Lines 416-420: The interpretation of the variance partitioning results is not quite accurate. The “interaction zones” (overlap of the diagram) are additive to the influence of a single factor. This means that the variation explained by sediment chemical properties alone was 6% and when an interaction with species was inferred it was 11%. Hence, the interaction doesn’t reduce the influence of the factors but adds another layer of information.

- **The interpretation has been updated and the needed corrections effected (Lines 418 – 426)**

**Discussion**

12. I would like to see a note in the discussion in which the authors discuss the potential effect of intertidal zonation on the microbiome. They show nicely how the tree species have an effect on the bacterial community, but fail to mention in the discussion, that the trees follow a strict zonation from sea to land. While the study is not designed to disentangle the question whether the trees or their intertidal position determine the microbiome more, this is an important point to mention. Especially as the sedimentary conditions have such a large influence too.

- **The discussion section has been updated to include the potential influence of mangrove zonation from sea to land on the bacterial communities (Lines 263 – 269 and lines 550 – 557).**
