# Supplementary material for: 16S rRNA gene amplicon-based metagenomic analysis of bacterial communities in the rhizospheres of selected mangrove species from Mida Creek and Gazi Bay, Kenya
Source: PLoS One. 2021 Mar 23;16(3):e0248485. doi: 10.1371/journal.pone.0248485 (PMC7987175; doi:10.1371/journal.pone.0248485)
Supplement: Attachment — Submitted filename: Responses to Reviewers.doc [file pone.0248485.s015.doc]

**Report on the responses raised by reviewer #4**

**PONE-D-20-21986R2**
16S rRNA gene amplicon-based metagenomic analysis of bacterial communities in the rhizospheres of selected mangrove species from Mida Creek and Gazi Bay, Kenya

**Responses to comments raised by Reviewer #4:**

I found my comments on the manuscript by Muwawa et al. addressed satisfyingly. Two minor issues with the wording may be resolved as suggested to ensure clarity of the study site and design. If these two sentences can be fixed, I am happy to recommend this mauscript for publication.

**Methods:**

1. Line 124-127: A suggestion: The mangrove forests in both sites display a similar zonation and mangrove species contribution among the four dominant species A. marina (30%), R. mucronata (25%), S. alba (15%), and C. tagal (10%), which contribute about 80% of the total mangrove formation in both forests.

- **Lines124-127 have been revised as suggested by the reviewer;**
- **The mangrove forests in both sites display a similar zonation and mangrove species contribution among the four dominant species *A. marina* (30%), *R. Mucronata* (25%), *S. alba* (15%) and *C. tagal* (10%), which contribute about 80% of the total mangrove formation in both forests.**

2. Lines 140-142: The sentences seem repetitive. You also use the word species in a ambivalent way, which makes it very hard to understand. I would suggest a very simple sentence such as: “From pure stands of S. alba, R. mucronata, C. tagal, and A. marina, one specimen (or tree) was selected per stand. The selected trees were spaced at least 10 m apart.”

- **Lines140-142 have been revised as recommended by the reviewer;**

**From pure stands of *S. alba,*** ***R. mucronata, C. tagal* and *A. marina,* one tree was selected per stand. The selected trees were spaced at least 10 m apart.**
